# Factors Associated With Dropout During Recruitment and Follow-Up Periods of a mHealth-Based Randomized Controlled Trial for Mobile.Net to Encourage Treatment Adherence for People With Serious Mental Health Problems

**DOI:** 10.2196/jmir.6417

**Published:** 2017-02-21

**Authors:** Kati Anneli Kannisto, Joonas Korhonen, Clive E Adams, Marita Hannele Koivunen, Tero Vahlberg, Maritta Anneli Välimäki

**Affiliations:** ^1^ Department of Nursing Science University of Turku Turku Finland; ^2^ Satakunta Hospital District Pori Finland; ^3^ Turku University of Applied Sciences Turku Finland; ^4^ Institute of Mental Health Division of Psychiatry University of Nottingham Nottingham United Kingdom; ^5^ Department of Biostatistics University of Turku Turku Finland; ^6^ Turku University Hospital Turku Finland; ^7^ Hong Kong Polytechnic University Hong Kong China (Hong Kong)

**Keywords:** text messaging, mobile health, antipsychotics, mental health, psychiatric services, methodological study

## Abstract

**Background:**

Clinical trials are the gold standard of evidence-based practice. Still many papers inadequately report methodology in randomized controlled trials (RCTs), particularly for mHealth interventions for people with serious mental health problems. To ensure robust enough evidence, it is important to understand which study phases are the most vulnerable in the field of mental health care.

**Objective:**

We mapped the recruitment and the trial follow-up periods of participants to provide a picture of the dropout predictors from a mHealth-based trial. As an example, we used a mHealth-based multicenter RCT, titled “Mobile.Net,” targeted at people with serious mental health problems.

**Methods:**

Recruitment and follow-up processes of the Mobile.Net trial were monitored and analyzed. Recruitment outcomes were recorded as screened, eligible, consent not asked, refused, and enrolled. Patient engagement was recorded as follow-up outcomes: (1) attrition during short message service (SMS) text message intervention and (2) attrition during the 12-month follow-up period. Multiple regression analysis was used to identify which demographic factors were related to recruitment and retention.

**Results:**

We recruited 1139 patients during a 15-month period. Of 11,530 people screened, 36.31% (n=4186) were eligible. This eligible group tended to be significantly younger (mean 39.2, SD 13.2 years, *P*<.001) and more often women (2103/4181, 50.30%) than those who were not eligible (age: mean 43.7, SD 14.6 years; women: 3633/6514, 55.78%). At the point when potential participants were asked to give consent, a further 2278 refused. Those who refused were a little older (mean 40.2, SD 13.9 years) than those who agreed to participate (mean 38.3, SD 12.5 years; t1842=3.2, *P*<.001). We measured the outcomes after 12 months of the SMS text message intervention. Attrition from the SMS text message intervention was 4.8% (27/563). The patient dropout rate after 12 months was 0.36% (4/1123), as discovered from the register data. In all, 3.12% (35/1123) of the participants withdrew from the trial. However, dropout rates from the patient survey (either by paper or telephone interview) were 52.45% (589/1123) and 27.8% (155/558), respectively. Almost all participants (536/563, 95.2%) tolerated the intervention, but those who discontinued were more often women (21/27, 78%; *P*=.009). Finally, participants’ age (*P*<.001), gender (*P*<.001), vocational education (*P*=.04), and employment status (*P*<.001) seemed to predict their risk of dropping out from the postal survey.

**Conclusions:**

Patient recruitment and engagement in the 12-month follow-up conducted with a postal survey were the most vulnerable phases in the SMS text message-based trial. People with serious mental health problems may need extra support during the recruitment process and in engaging them in SMS text message-based trials to ensure robust enough evidence for mental health care.

**ClinicalTrial:**

International Standard Randomized Controlled Trial Number (ISRCTN): 27704027; http://www.isrctn.com/ISRCTN27704027 (Archived by WebCite at http://www.webcitation.org/6oHcU2SFp)

## Introduction

Serious mental health problems are a major problem around the world [1]. They are associated with cognitive deficits, such as distortions in thinking or troubles in paying attention or working memory [2], a lack of treatment adherence [3], rehospitalization [4], and lifelong disability [5]. Mobile technology has become a popular way to deliver interventions to facilitate adherence to chronic disease management [6], including to people with serious mental health problems [7-9].

Recently, interventions with mobile phone technology (mHealth) have been applied to randomized controlled trials (RCTs) [10]. However, many methodological concerns in RCTs have been raised when mHealth interventions have targeted people with serious mental health problems [9]. Most concerns are related to inadequately reported details in the studies [10], such as the patient recruitment process [9], participant engagement in mHealth-based interventions [11], or technological details related to intervention delivery (eg, amount of undelivered text messages or patients changing a phone number) [12]. Because the mental health area continues to adopt new technologies in clinical practice [7,13,14], generating high-quality research is essential.

On the other hand, conducting mHealth-based research among people with mental health problems includes challenges [11]. First of all, reaching the target group may be difficult [11,15]. Patients may refuse to participate due to a fear of or suspicious thoughts about mobile devices [16]. They may also distrust the credibility of a mHealth intervention [11]. Other concerns related to mHealth use are user privacy, confidentiality, and online security [11]. People with serious mental health problems may be “digitally divided,” which makes them vulnerable for not benefiting from mHealth services [17]. For example, not everyone is willing to use mobile technology [13], has access to technology [17,18], or is skilful or familiar with mobile phones [19]. Further, low engagement and discontinuation are fundamental problems in technology-based intervention studies [9,20]. The participants may stop the intervention because they feel that it is too complex, time consuming [11], or repetitive and, therefore, the intervention becomes a mere routine.

To better understand how mobile apps could be developed, evaluated, and implemented into routine care, it is important to truly understand which study phases make the RCT the most vulnerable in the field of mental health care. Still, many important parts of the study methodology are inadequately reported in RCTs, particularly regarding interventions targeting people with serious mental health problems [9]. Therefore, we mapped the recruitment and the 12-month trial follow-up periods in order to provide a picture of the dropout predictors from a mHealth-based multicenter RCT, titled “Mobile.Net” (ISRCTN: 27704027). Recruitment and engaging participants in trials involving psychosis is problematic; there are numerous ways in which it can go wrong (eg, consenting, attrition). This paper describes a case study of recruitment and follow-up processes, and problems in this context, based on the Mobile.Net trial. Multiple regression was used to identify which demographic factors are related to recruitment and retention. The main results of the Mobile.Net trial will be reported elsewhere.

## Methods

### Mobile.Net Trial

Mobile.Net is a nationwide multicenter randomized controlled two-armed trial. The Mobile.Net trial evaluated the effects of tailored short message service (SMS) text messages constructed to encourage patient medication adherence and outpatient care for adult patients with psychosis [21]. Participants in the intervention group received semiautomatic text messages for 12 months (approximately 10 per month; range 2-25 text messages) based on their preferences [22]. They were able to decide the amount, timing, and frequency of the SMS text messages delivered. They were also able to change the content or timing of the messages during the trial. Treatment as usual was offered to all participants. The study was carried out according to the Declaration of Helsinki and approved by the Ethics Committee of the Hospital District of Southwest Finland. Written informed consent was obtained from the study participants after they were given a complete description of the study.

### Population

There were a total of 1139 participants, men and women, ranging in age from 18 to 65 years. Each participant had a continuing prescription for antipsychotic medication, access to a mobile phone, and the ability to use the Finnish language. After participants were recruited, they were then randomized. Forensic patients and those having a planned nonacute treatment period were excluded from the study [21].

### Procedure

Recruitment, including activities conducted before and during participant enrollment [23], occurred face-to-face in 45 psychiatric hospital wards in Finland (between September 5, 2011 and November 30, 2012). Research nurses in each study ward performed chart reviews to check the eligibility of each patient admitted to the study ward [24]. After completion of the baseline data, patients were allocated randomly (computer-based randomization with four block randomization) into two groups (SMS text message intervention group or control group) [21].

Attrition, including actions after enrollment in the study [23], was assessed at the 12-month follow-up period (between September 5, 2012 and December 31, 2013). Attrition was defined as the loss of eligible participants from the study groups [25]. To gather follow-up data, a postal survey, including the Quality of Life Enjoyment and Satisfaction Questionnaire (Q-LES-Q) by Endicott et al [26] and the Client Satisfaction Questionnaire (CSQ-8) by Atkisson and Greenfield [27], was carried out. Follow-up data were collected by members of the research group. During the study period and follow-up, each point of contact with participants was completely tracked and recorded. Attrition was assessed during the intervention and during the follow-up.

### Measures

Data for this paper were divided into two categories: data concerning patient recruitment and data relating to attrition (Textbox 1).

**Measures concerning patient recruitment and attrition from the study.**

**Recruitment** (variable: measurement)

1) Screened: (n)

2) Eligible: n (%)

3) Consent not asked: n (%)

4) Refused: n (%)

5) Enrolled: n (%)

Recruitment speed: n/day

**Attrition** (variable: measurement)

1) During SMS intervention: dropout rate

2) During follow-up

- Telephone interview: dropout rate
- Participant’s notification: dropout rate
- Postal survey: dropout rate
- Register data retrieval: dropout rate

Recruitment data were categorized into five groups: (1) patients screened for eligibility, (2) eligible participants, (3) eligible participants whose consent was not requested, (4) participants who refused to participate at the point of contact in the psychiatric ward, and (5) those who consented to participate. Outcomes were recorded as screened, eligible, consent not asked, refused, and enrolled. In addition, to track the pace of recruitment, a record of all identified, screened, eligible, unwilling, and successfully recruited patients was kept using a specific monitoring sheet developed for the trial. Patient flow was monitored and recorded daily on the study wards. Daily progress of patient recruitment was reported as “recruitment pace” (ie, how many new patients were recruited e each day) [28].

Attrition data were categorized into two groups: (1) attrition during SMS text message intervention and (2) attrition during the 12-month follow-up period [29]. Attrition during the follow-up was then divided into four categories, based on the follow-up data collection method: (1) telephone interview, (2) participants’ notification (ie, withdrew from the follow-up survey), (3) postal survey, or (4) register data retrieval from the Finnish National Care Register for Health Care [30]. Outcomes were reported as dropout rates. In our study, the dropout rates were calculated based on telephone or postal survey responses, or by whose data were not available in register data retrieval due to an incorrectly entered ID.

### Statistical Analysis

Descriptive statistics (frequency, percentage, mean, standard deviation) were used to describe participants’ demographic characteristics, recruitment, and attrition metrics (study participants lost in the follow-up). The demographic variables examined included age, gender, marital status (lives alone, ie, single, divorced, or widowed; lives with someone, ie, married), vocational education (none, vocational education), employment status (employed/self-employed, retired, student, job seeker), diagnosis (*International Statistical Classification of Diseases and Related Health Problems, Tenth Revision* [31]), and age at first contact with psychiatric services. To analyze possible differences between patients who participated in the study and those who dropped out, *t* tests and chi-square tests were used.

Multiple logistic regression analysis was used to determine predictors of dropping out of the 12-month postal survey follow-up. Participants’ demographic characteristics, including age, gender, marital status, vocational education, and employment status, were chosen as predictors and added to the analysis [8,32]. All data were analyzed using SPSS version 21. A *P* value <.05 was interpreted as a statistically significant difference.

## Results

### Recruitment

A total of 11,530 patients admitted within psychiatric inpatient hospital wards were screened during the 15-month (453 days) recruitment period. There were 6565 who did not meet the eligibility criteria. A total of 779 patients dropped out before the eligibility assessment because they were transferred to another ward or rapidly discharged from hospital. Of the candidates who were screened, 36.31% (4186/11,530) appeared eligible.

Of the 4186 eligible patients, informed consent was asked from 3417 (81.63%). Informed consent was not asked in 18.37% (769/4186) of the cases because the person was quickly discharged from the ward, absconded from hospital, or the research nurses simply forgot to ask.

When age and gender of the screened noneligible and eligible patients were compared, it was found that the eligible patients were generally younger than the noneligible patients (*P*<.001). Men were more often noneligible than women (*P*<.001) (Table 1).

**Table 1:** Demographic characteristics comparable across all stages.

Stage of study		N	Age (years)				Gender (male)		
			Mean (SD)	Range	*t* (df)	*P*	n/N (%)	χ^2^_1_	*P*
**Screened**		11,530	41.1 (14.6)	16-90	–9.86 (3492)	<.001	6164/11,461 (53.78)		
	Noneligible	6565	43.7 (16.1)	16-90			3633/6514 (55.77)	30.7	<.001
	Eligible	4186	39.2 (13.2)	18-65			2103/4181 (50.30)		
**Point of consent**					3.23 (1842)	.001		0.3	.59	
	Refused	2278	40.2 (13.9)	18-65			1142/2274 (50.22)		
	Randomized	1139	38.3 (12.5)	18-65			560/1139 (49.17)		
**Intervention period**					–0.73 (561)	.47		7.2	.009
	Completers	536	38.5 (12.7)	18-65			261/536 (48.7)		
	Dropouts	27	40.3 (13.0)	21-63			6/27 (22.2)		
**Follow-up period**					–1.28 (36)	.21		10.1	.002
	Completers	1088	38.3 (12.5)	18-65			545/1088 (50.09)		
	Withdrawals	35	41.1 (12.6)	18-63			8/35 (22.9)		
**Postal survey**					–8.14 (1120)	<.001		18.5	<.001
	Completers	534	41.5 (12.6)	18-65			227/534 (41.0)		
	Dropouts	589	35.5 (11.8)	18-65			326/589 (59.0)		

Out of the 3417 eligible participants whose consent was asked, 2278 patients (66.67%) refused to participate in the study. Although reasons for refusal were not asked due to ethical guideline requirements [33], some patients voluntarily offered explanations, such as they did not know how to use mobile phones or text messages, their mobile phone was broken, or they did not have a mobile subscription at the that time. Patients who refused to participate were older than consenting, randomized patients (*P*=.001) (Table 1).

The pace of recruitment was analyzed based on the number of new patients recruited each day. At the beginning of the study, recruitment was slow. The recruitment rate reached its peak 15 months after enrollment started. For every 10 screens completed, one person was successfully enrolled, at an average recruitment speed of 76 participants each month (2.5 participants per day).

Of the 1139 patients who were enrolled in the study, the data of 16 participants were excluded due to either the withdrawal of informed consent (n=10), the patient did not meet the inclusion criteria (n=5), or a recruitment error (n=1). This left us with a total of 1123 participants (intervention group: n=563; control group: n=560).

### Attrition

#### Attrition During SMS Text Message Intervention Period

A total of 569 eligible participants were allocated to a group to receive tailored SMS text messages for 12 months. The data of six participants were excluded from the analyses due to either a lack of written informed consent (n=2), the patient did not meet the inclusion criteria (n=3), or an erroneous randomization to study group (n=1). This left us 563 participants.

Of the 563 participants who received text messages, 27 dropped out during the 12-month intervention period (4.8%). In cases where a patient did not want to continue with the text message intervention, the researchers were notified by the participant, a relative, or a research nurse. Three participants dropped out before the intervention even began, and 24 within the 12-month intervention period [22]. Participants who dropped out during the intervention were still included in the study [34]. We observed that intervention dropouts were more often women than men (χ^2^_1_=7.2, *P*=.009), but found no other statistically significant differences (Multimedia Appendix 1).

#### Attrition During the 12-Month Follow-Up Period

Information about participants who dropped out after the 12-month follow-up was divided into four categories based on the data collection method: (1) telephone interview, (2) participants’ notification (ie, withdrew from the follow-up survey), (3) postal survey, or (4) register data retrieval.

First, telephone interviews (for the intervention group only) were conducted after the 12-month text message intervention to explore participants’ feedback on the text message service (n=569). We attempted to reach 558 participants by telephone for an interview; after the telephone calls were made, we had 403 completed questionnaires (response rate 72.2%, 403/558) [35]. The dropout rate from these telephone interviews was 27.8% (155/558). Dropouts were younger, usually men, without a vocational education, and were also younger at the time of first contact with psychiatric services compared with those who completed the questionnaire [35].

Second, 35 participants expressed that they wanted to withdraw from the follow-up surveys (intervention group: 5.5%, 31/563; control group: 0.7%, 4/560; χ^2^_1_=21.4, *P*<.001). Follow-up surveys were not conducted with these participants, but their register data were retrieved. The dropout rate regarding follow-up surveys was 3.12% (35/1123). Women requested to withdraw from the follow-up surveys more often than men did (χ^2^_1_=10.1, *P*=.002). We found no other statistically significant differences (Multimedia Appendix 1).

Third, a postal survey (n=1123) was conducted after the 12-month study period to measure participants’ quality of life (Q-LES-Q [26]) and satisfaction with the treatment (CSQ-8 [27]). Of 1123 participants, 589 did not return the postal survey (dropout rate: 52.45%, 589/1123). There was a statistically significant difference in demographic characteristics between postal survey completers (ie, returned the completed postal survey) and dropouts (Multimedia Appendix 1).

Fourth, register data retrieval was conducted after the 12-month follow-up period. Out of 1123 participants, the register data of four participants were not available from the Finnish National Care Register for Health Care [30], making the dropout rate 0.36%. Demographic characteristics of participants whose register data were available will be reported elsewhere.

#### Factors Related to Patient Attrition

Through a logistic regression analysis, Table 2 illustrates the associations between participants’ demographic characteristics (age, gender, marital status, vocational education, and employment status) and risk of dropping out from the postal survey. Odds ratios were not estimated from other variables due to missing data. Participants’ age, gender, vocational education, and employment status seemed to predict their risk of dropping out from the postal survey. The participants in this group were older, more often women, had a vocational education, and were more often retired.

**Table 2:** Associations between participants’ demographic characteristics and risk of dropping out of the postal survey (N=1123).

Demographic characteristics		OR (95% CI)	*P*
**Age**		0.96 (0.95-0.97)	<.001
**Gender**			
	Female	1	<.001
	Male	1.63 (1.27-2.11)	
**Marital status**			
	Lives with someone	1	.46
	Lives alone	1.12 (0.83-1.50)	
**Vocational education**			
	Vocational education	1	.04
	None	1.37 (1.01-1.84)	
**Employment status**			
	Student	1	.001
	Employed/self-employed	1.65 (1.01-2.70)	.045
	Retired	2.29 (1.45-3.61)	<.001
	Job seeker	2.44 (1.50-3.97)	<.001

## Discussion

### Principal Results

The results of this study demonstrate that it was challenging to recruit, and engage, participants in an SMS text message-based trial follow-up. One-third of patients (36.31%) appeared eligible, and two-thirds of eligible patients (66.67%) refused to participate. Participants were well engaged with the SMS text message intervention provided, but their engagement with the trial follow-up varied: the highest being with the register data retrieval (99.64%) and lowest with the postal survey (47.55%). Participants’ demographic characteristics (age, gender, vocational education, and employment status) were seen as dropout predictors.

In our study, within the context of psychiatric inpatient care, we were able to recruit 1139 individuals (33.33%) out of 3417 eligible participants, whose consent was requested. Age and gender tended to be factors influencing recruitment and refusal. Our refusal rate was 66.67% (2278/3417), which is in line with previous studies suggesting that high refusal rates are a major problem faced during the recruitment process [11,15]. Patients’ illness-related issues [14,19] and perceived stigma were seen as barriers to participation in previous studies [36,37]. This may also be the case in our study, which focused on people treated with antipsychotic medication recruited from hospital wards providing psychiatric care.

Lack of interest in the trial [11], lack of motivation to use mobile technology-based interventions [13], or a lack of capacities may also have affected the refusal rates in our trial. During the participant screening, some patients expressed their inability to use mobile phones or text messaging, stating that as the reason for their refusal. This was unexpected because mobile phone text messages have been proven to be feasible and acceptable among people with severe mental health problems [38], as well as easy to use [35]. Therefore, our findings may reveal that a digital divide may still exist in the area of mental health, although relevant literature identifies this concept as promising [14]. Previous literature also shows that people with mental health problems are able and willing to use mobile-based interventions when given the opportunity [38,39]. In our case, it might have been useful to train those who said they did not know how to use mobile phones, and to offer mobile phones to those who did not have them. This might have given us important knowledge about the digital divide, especially those who are not so familiar with the mobile technology.

The attrition rate during the SMS text message intervention was low (4.8%). This finding does not support previous findings stating that low engagement and discontinuation are major problems in intervention studies [11,14]. However, it is possible that participants who stopped using the SMS text message intervention did not notify the researchers. What we found here was that women dropped out from the SMS text message intervention more often than men did. This is contradictory to the results of Ben-Zeev and colleagues [8], who found that women were significantly more engaged with mHealth interventions than men were. Further, participants’ demographic characteristics (age, gender, vocational education, and employment status) predicted the risk of leaving the study early and, subsequently, not participating in the follow-up. This is in line with previous studies, which reported that participants who left the studies early differed from those who were retained [8,32]. Therefore, it is important to identify feasible and useful mHealth interventions targeting different patient groups and, further, to identify factors that facilitate or prevent patient engagement with mHealth interventions.

Patient engagement in the trial during the follow-up varied depending on the source of data collected, the highest being in the register data retrieval (99.64%) and the lowest in the postal survey (47.55%). Low engagement and discontinuation have been found as fundamental problems in technology-based intervention studies [9,20]. Participants’ age, gender, vocational education, and employment status seemed to predict their risk of dropping out from the postal survey. This group of older, women, and retired participants may also reveal the digital divide in this patient group. It may be that they just do not value technology as much as younger people do and, therefore, did not participate in the postal survey. Recently, however, technology usage has increased among older adults [40]. This is promising and may give researchers new hints on how to engage participants in trials. Given this, it is important to consider which data collection methods are appropriate to use among people with serious mental health problems, and to identify factors that facilitate patient engagement in mHealth-based trial follow-ups.

### Limitations

Our study has some limitations. The recruitment data concerning information about screened patients lacked some information, especially about patients’ ages. Therefore, results related to patient demographics concerning recruitment have to be handled with caution. More importantly, we did not gather knowledge about the participants’ actual SMS text message use. Therefore, we lack knowledge about participants’ true engagement with the SMS text message intervention. However, according to our findings before the study actually started, participants were very satisfied with the intervention [35].

A key strength of this study was in its large nationwide sample of people treated with antipsychotic medication. Another was that, to the best of our knowledge, this was the largest trial evaluating a text message system. Our findings regarding attrition are important for those conducting similar RCTs among people with severe mental health problems, although this group may well have different issues with the technology when compared with others [17,41].

### Conclusions

Initial patient recruitment and then engagement in the 12-month postal survey follow-up were the most vulnerable phases in the SMS text message-based trial. This may indicate that people with serious mental health problems may need extra support during the recruitment process, and necessitate further support to engage in completion of these follow-up questionnaires—at least within SMS text message trials.

Researchers should acknowledge the possible digital divide for people with serious mental health problems, and choose convenient and efficient data collection methods for study follow-ups. At follow-up, for Mobile.Net, high-grade routine data were almost complete. Methods of trials should take much more consideration of the nature of the target group of participants; otherwise, evidence is dogged with high attrition with the accompanying speculation of researchers. No statistical technique or learned speculation can make up for loss to follow-up. The solutions are likely to vary for different client groups. We think more research is needed both to investigate the support of the recruitment process and methods of follow-up in technology-based RCTs. Asking people to complete forms that are likely to result in grossly incomplete datasets could be considered an unethical—and potentially dangerous—waste of time and resources.

## Abbreviations

CSQ-8: Client Satisfaction Questionnaire
Q-LES-Q: Quality of Life Enjoyment and Satisfaction Questionnaire
RCT: randomized controlled trial
SMS: short message service

## Appendix

Multimedia Appendix 1 Additional demographic characteristics comparable across latter stages of study.
