# Supplementary material for: Factors Associated With Dropout During Recruitment and Follow-Up Periods of a mHealth-Based Randomized Controlled Trial for Mobile.Net to Encourage Treatment Adherence for People With Serious Mental Health Problems
Source: J Med Internet Res. 2017 Feb 21;19(2):e46. doi: 10.2196/jmir.6417 (PMC5340923; doi:10.2196/jmir.6417)
Supplement: Multimedia Appendix 1 [file jmir_v19i2e46_app1.pdf]

Supplementary Table 1. Additional demographic characteristics comparable across latter stages of study.

Demographic characteristics	Intervention period				Follow-up period				Postal survey			
	Completers (n=536)	Dropouts (n=27)	*t* or χ^2 (*df*)[a]	*P*	Completers (n=1088)	Withdrawals (n=35)	*t* or χ^2 (*df*)[a]	*P*	Completers (n=534)	Dropouts (n=589)	*t* or χ^2 (*df*)[a]	*P*
**Age (years), mean (SD)**												
Current	38.5 (12.7)	40.3 (13.0)	–0.73 (561)	.47	38.3 (12.5)	41.1 (12.6)	–1.28 (36)	.21	41.5 (12.6)	35.5 (11.8)	–8.14 (1120)	<.001
First contact	27.3 (11.6)	27.7 (12.6)	–0.17 (554)	.86	27.1 (11.5)	28.7 (13.3)	–0.76 (1104)	.45	28.9 (12.1)	25.6 (10.9)	–4.65 (1058)	<.001
**Gender (male), n (%)**	261/536 (48.7)	6/27 (22.2)	7.2 (1)	.009	545/1088 (50.09)	8/35 (23)	10.1 (1)	.002	227/534 (41.0)	326/589 (59.0)	18.5 (1)	<.001
**Marital status, n (%)**			0.5 (1)	.46			0.71 (1)	.33			9.6 (1)	.002
Lives alone	389/532 (73.1)	18/27 (66.7)			793/1083 (73.22)	23/35 (66)			366/533 (68.7)	450/585 (76.9)		
Lives with someone	143/532 (26.9)	9/27 (33.3)			290/1083 (26.78)	12/35 (34)			167/533 (31.3)	135/585 (23.1)		
**Vocational education, n (%)**			0.9 (1)	.34			0.3 (1)	.58			23.8 (1)	<.001
None	169/529 (31.9)	6/26 (23.1)			333/1075 (30.98)	9/34 (27)			126/530 (23.8)	216/579 (37.3)		
Vocational education	360/529 (68.1)	20/26 (76.9)			742/1075 (69.02)	25/34 (73)			404/530 (76.2)	363/579 (62.7)		
**Employment status, n (%)**			4.2 (3)	.25			4.6 (3)	.20			13.1 (3)	.005
Employed/self-employed	112/527 (21.3)	9/27 (33.3)			221/1070 (20.65)	10/35 (29)			123/520 (23.7)	108/585 (18.5)		
Retired	253/527 (48.0)	14/27 (51.9)			519/1070 (48.50)	20/35 (57)			261/520 (50.2)	278/585 (47.5)		
Student	57/527 (10.8)	2/27 (7.4)			123/1070 (11.50)	2/35 (5)			59/520 (11.3)	66/585 (11.3)		
Job seeker	105/527 (19.9)	2/27 (4.9)			207/1070 (19.35)	3/35 (9)			77/520 (14.8)	133/585 (22.7)		
**Diagnoses (*ICD-10*),[b] n (%)**			2.6 (5)	.76			5.1 (5)	.41			14.6 (5)	.01
F10-F19[a]	29/507 (5.7)	2/25 (8.0)			58/1020 (5.69)	2/29 (7)			20/497 (4.0)	40/552 (7.2)		
F20-F29[b]	203/507 (40.0)	8/25 (32.0)			409/1020 (40.10)	8/29 (28)			218/497 (43.9)	199/552 (36.1)		
F30-F39[c]	152/507 (30.0)	10/25 (40.0)			311/1020 (30.49)	14/29 (48)			160/497 (32.2)	165/552 (29.9)		
F40-F49[d]	45/507 (8.9)	2/25 (8.0)			85 /1020 (8.33)	2/29 (7)			34/497 (6.8)	53/552 (9.6)		
F60-F69[e]	69/507 (13.6)	2/25 (8.0)			132/1020 (12.94)	2/29 (7)			55/497 (11.1)	79/552 (14.3)		
Others	9/507 (1.8)	1/25 (4.0)			25/1020 (2.45)	1/29 (3)			10/497 (2.0)	16/552 (2.9)		

[a] For age categories, *t* test was used. For all others, it was chi-square.

[b] *ICD-10*: *International Statistical Classification of Diseases and Related Health Problems Tenth Revision*; F10-F19: Mental and behavioral disorders due to psychoactive substance use; F20-F29: schizophrenia, schizotypal, and delusional disorders; F30-F39: mood (affective) disorders; F40-F49:neurotic, stress-related, and somatoform disorders; F60-F69: disorders of adult personality and behavior.
